# Editorial: Bacteriophage and host interactions

**DOI:** 10.3389/fmicb.2024.1422076

**Published:** 2024-05-31

**Authors:** Sylwia Bloch, Alicja Wegrzyn

**Affiliations:** ^1^Department of Molecular Biology, University of Gdansk, Gdansk, Poland; ^2^University Center for Applied and Interdisciplinary Research, University of Gdansk, Gdansk, Poland

**Keywords:** bacteriophages, phage-host interactions, phage therapy, lytic and lysogenic cycles, pathogenic bacteria

Bacteriophages (shortly phages) are defined as viruses that infect bacteria. They play a crucial role in many ecosystems by affecting the bacterial communities (Dion et al., 2020). This phenomenon is tightly bound with the fact that phages have complex interactions with their hosts. They are unable to carry out most of the biological processes on their own and need a live bacterial cell to multiply efficiently. As a result, phages have an influence on host abundance, diversity, physiology, and metabolism. For these reasons, bacteriophages are considered as a drivers of microbial diversity and may be use as means to understand many molecular mechanisms in biological processes. Over the last two decades, phages have been also intensively studied for their usage in biotechnology, medicine, food industry, and agriculture (Naureen et al., 2020; Albrycht et al., 2022). Application of bacteriophages in many areas of life undoubtedly requires, for safety reasons, a deep analysis of complex phage-bacteria interactions.

This Research Topic has been devoted to works that aim to help researchers understand the network of various interactions between bacteriophages and their hosts. The current Research Topic consists of fourteen articles. These articles are extremely interesting and well-representing the diversity of the studies on biology and biotechnology of bacteriophages.

Phages develop some strategies to adapt to different environmental conditions (Dennehy and Abedon, 2020). In the first article, Laguno-Castro et al. demonstrated that the adaptive pathway of phage Qβ, in the face of similar variations of *Escherichia coli* cells density, depends on temperature. They showed that in the conditions of the reduction of host availability, Qβ chooses the same adaptive strategy at 30 and 43°C, which is related to the improvement of its entry into bacterial cell. The authors concluded that the adaptation of Qβ to low bacterial cells availability involves different mutational pathway depending on the balance between fitness advantages and costs of replication at each temperature.

Through a series of interactions between phage-encoded receptor binding proteins (RBPs) and receptors located on the cell surface, the virus recognizes a sensitive bacteria (Bertozzi Silva et al., 2016). The work by Forrest et al. presented the evidence of genetic dependencies between *sap, csaB* and the sporulation proteins Spo0A, Spo0B, and Spo0F of *Bacillus anthracis* with the RBPs of the AP50c and W1 bacteriophages. Thanks to these results, we understood better the mechanisms of attachment and entry strategies of phages infecting the *Bacillus anthracis* bacterium.

One of challenges of molecular engineering is how to introduce foreign DNA to environmental bacterial cells. An excellent solution to this problem is the P1 bacteriophage (Westwater et al., 2002). The third article, published by Giermasińska-Buczek et al., focused on the interactions of P1 with an environmental epiphytic isolate *Pantoea agglomerans* L15. The authors demonstrated that the L15 can be a host for the phage P1. Moreover, based on analysis of the L15 interaction with the P1 *c1-100* Tn9 mutant, they have discovered the influence of antibiotic selection pressure on overcoming the barriers protecting bacterial cells from foreign DNA.

Bacteria evade viral infections by developing various defense systems (Egido et al., 2022). In the review article, Akritidou and Thurtle-Schmidt described the composition, structure, and function of overcoming lysogenization defect (OLD) nucleases that contain an ABC ATPase and Toprim domains. The authors also highlighted the role of OLD proteins in the anti-phage defense systems, such as the Gabija system and retrons.

Our understanding of phage-host interactions is extremely important to effectively use bacteriophages against pathogenic bacteria in a wide range of sectors of our life. Several articles in the Research Topic addressed this issue. In the review article, Glizniewicz et al. presented different phage-mediated strategies for combating polymicrobial biofilms. They also summarized the advantages and disadvantages of phage therapy, and familiarized the reader with the perspectives of such form of treatment.

The next article related to phage therapy, published by Pacios et al., focused on improving the lytic activity of phage vB_KpnS_VAC35 against two bacteremia-causing isolates of *Klebsiella pneumoniae*. The authors demonstrated that bacterial cells exposed to mucin and the *N*-acetyl cysteine exhibited a significant reduction in the frequency of phage resistance compared to mucin-treated bacteria. Determining the dependences between the mucoid environment and the difficulties in applying phage therapy allowed researchers to create a promising approach based on the application of mucolytic agents together with phages against *K. pneumoniae* infections.

In turn, the work by Chen et al. described the identification and characterization of phage vB_KpP_HS106 which infects 26 *K. pneumoniae* strains isolated from dairy farms in Shanghai. The great advantages of this virus are a good tolerance to extreme environments and the ability to efficient reduction of *K. pneumoniae* in milk and chicken meat. Therefore, HS106 has a prospects as biocontrol agent used against *K. pneumoniae* in foods.

Another phage with antimicrobial potential has been presented by Cui et al.. The vB_PaeP_ASP23 infects *Pseudomonas aeruginosa* strain L64 which causes hemorrhagic pneumonia in minks. Interestingly, the authors also confirmed the lytic activity of lysin and holin of the ASP23 against some tested Gram-negative and -positive bacteria, respectively. In this case, all three, ASP23, LysASP and recombinant phage HolASP can be considered as promising antimicrobial tools in the mink farming industry.

In the next article, Zou et al. described a novel phage MA9V-1 infecting *Chryseobacterium indologenes* which is the primary agent of root rot of *Panax notoginseng*. MA9V-1 exhibited a quick adsorption rate (>75% in 8 min) and a brief latent period (20 min). Moreover, the phage particles were stable in a relative broad range of temperatures (30–60°C). The authors suggested that these features of MA9V-1 make this virus promising in its use for preventing the diseases of medical plants.

Other studies on the control of plant diseases were presented in the article by Borowicz et al.. The researchers characterized the dickeyocin P2D1of *Dickeya dadantii* strain 3937, a representant of plant pathogenic Soft Rot *Pectobacteriaceae* (SRP). Interestingly, P2D1 could kill eight different *Dickeya* ssp. Moreover, this tailocin was stable at temperatures between 4 and 50°C, in pHs ranking from 3.5 to 12, and in osmotic conditions generated by NaCl (0.01–1 M). All of these features indicate that the newly identified dickeyocin would be a grate tool in controlling SRP infections in crops.

One of the biggest challenges in designing bacteriophage-based therapy is to identify the most appropriate phage which will be effective in the fight against pathogenic bacteria. A promising solution to this problem may be the water-in-oil droplet-mediated method described in the work by Hoshino et al.. This approach is based on the combination of droplet technology with a fluorescent YOYO-1 dye that stains the virus particles. Such an innovative method will enable researchers to isolate previously unidentified viruses for known host when applied to environmental samples.

Quite different approach was presented in the article by Aggarwal et al.. The authors have developed an *in-silico* tool, named PhageTB, for the prediction of phage-bacteria interactions with high accuracy. Interestingly, this bioinformatics tool contains three modules: host for phage, phage-host interaction and phage for host. We believe that PhageTB will be an effective method for prediction of phage-based therapy against bacterial infections.

It should also be mentioned that the pathogenicity of some bacteria is related to the presence of prophages in their genomes. In the next article, Mangieri et al. characterized inducible prophages from three shiga toxin producing *E. coli* strains. Interestingly, the authors indicated that some factors in cheese making process, such as NaCl and lactic acid, can be considered as potential stressors to induce phage release. Unfortunately, such a phenomenon promotes the shiga toxins transmission among bacteria and affects the safety of the cheese manufacturing.

In turn, the work by Li et al. described the role of spontaneous induction of newly identified prophage phi485 in avian pathogenic *E. coli* strain DE458. This study reveals that the deletion of phi458 leads to a strong decreased of biofilm formation and increased colonization abilities and virulence of DE458Δphi458. Undoubtedly, this report will allow us to better understand the relationship between the spontaneous induction of prophages and the pathogenicity of *E. coli* bacteria.

This Research Topic of articles includes articles describing a number of new and interesting phenomena regarding the complex networks of various dependencies between bacteriophages and their hosts. Readers are encouraged to get acquainted with details of the works published in the frame of this Research Topic as they provide a very valuable knowledge about pathogenic bacteria, phages and their potential applications in many areas of our life.

## Author contributions

SB: Writing—original draft. AW: Writing—review & editing.
